# Examining the Correlates of Adolescent Food and Nutrition Knowledge

**DOI:** 10.3390/nu13062044

**Published:** 2021-06-15

**Authors:** Rachel Brown, Jamie A. Seabrook, Saverio Stranges, Andrew F. Clark, Jess Haines, Colleen O’Connor, Sean Doherty, Jason A. Gilliland

**Affiliations:** 1 Department of Epidemiology and Biostatistics, The University of Western Ontario, London, ON N6A 3K7, Canada; rbrow49@uwo.ca (R.B.); jseabro2@uwo.ca (J.A.S.); saverio.stranges@uwo.ca (S.S.); 2 Human Environments Analysis Laboratory, The University of Western Ontario, London, ON N6A 3K7, Canada; aclark2@uwo.ca (A.F.C.); colleen.oconnor@uwo.ca (C.O.); sdoherty@wlu.ca (S.D.); 3 School of Food and Nutritional Sciences, Brescia University College, London, ON N6G 1H2, Canada; 4 Department of Paediatrics, The University of Western Ontario, London, ON N6A 3K7, Canada; 5 Children’s Health Research Institute, London, ON N6C 4V3, Canada; 6 Lawson Health Research Institute, London, ON N6C 2R5, Canada; 7 Department of Family Medicine, The University of Western Ontario, London, ON N6A 3K7, Canada; 8 Department of Medicine, The University of Western Ontario, London, ON N6A 3K7, Canada; 9 Department of Population Health, Luxembourg Institute of Health, L-1445 Strassen, Luxembourg; 10 Department of Geography & Environment, The University of Western Ontario, London, ON N6A 3K7, Canada; 11 Department of Family Relations and Applied Nutrition, University of Guelph, Guelph, ON N1G 2W1, Canada; jhaines@uoguelph.ca; 12 Department of Geography and Environmental Studies, Wilfrid Laurier University, Waterloo, ON N2L 3C5, Canada; 13 School of Health Studies, The University of Western Ontario, London, ON N6A 3K7, Canada

**Keywords:** adolescent health, knowledge, attitudes, practice, health literacy, quantitative evaluation, education

## Abstract

Food literacy is a set of skills and knowledge that are integral to diet. It is common among teenagers to not have basic food literacy skills needed to consume a healthy diet. This study examined: (1) the current state of food and nutrition knowledge among adolescents 13–19 years of age in the census metropolitan area of London, ON, Canada; and (2) correlates of food knowledge and nutrition knowledge among adolescents. Data for this study were drawn from baseline youth and parent survey data collected from a larger population health intervention study. Statistical analysis of the survey data indicates that higher parental education and higher median neighbourhood family income, the use of mobile health applications, liking to cook, as well as confidence in reading and understanding food labels were all consistently associated with increased food and nutrition knowledge. Findings may help guide future research towards optimal methods for delivering food literacy interventions to effectively educate teenagers. Results of this study may help guide policy makers, researchers, and public health professionals in developing appropriate food and nutrition programs and curriculums to combat the decline in food literacy skills.

## 1. Introduction

Research suggests that diet quality decreases in adolescence and remains suboptimal into adulthood [1]. Teenagers’ dietary patterns are characterized by meal skipping, frequent snacking, and high intakes of fast food [2,3]. Yet, teenagers often lack a sense of urgency for taking care of their health, as they struggle to conceptualize how their decisions in the short-term affect their future and long-term health [4]. This is concerning because dietary habits established in childhood and adolescence set the foundation for lifelong practices [5]. Poor eating habits and food behaviours during adolescence can also increase the risk for obesity and related chronic diseases in adulthood [2,5,6].

The ever-growing prevalence of fast-food outlets and large-scale food retail stores [7], as well as processed and convenience foods [3,8,9], has made high-calorie and nutrient-poor foods and beverages more readily available and affordable [7]. Parallel to these changes in our food environment is a growing acceptance and normalization of these unhealthy food options, possibly because people want food products that require little to no preparation [3,5,10]. Consequently, these readily available processed foods are diminishing the need for people to understand how to plan and prepare food for themselves [3,5,8,11,12]. In fact, Moubarac et al. [13] found that Canadians are consuming approximately 50% of their energy from ultra-processed food.

Eating habits and food behaviours are influenced by a host of inter-related factors [10]. Food and nutrition knowledge are critical factors that help adolescents navigate the complex food system, including purchasing healthy food options for preparing healthy meals, to understanding what is in our food, to how nutrients affect the body [7,11,14,15]. However, teenagers often have insufficient food and nutrition knowledge to select and consume a healthy diet [11,12,15,16]. The acquisition of food and nutrition knowledge is determined by a complex interplay of barriers and facilitators at various levels of influence that ultimately affect the dietary behaviours of adolescents. For example, sociodemographic factors, such as education, income, ethnicity, and gender are intrapersonal variables that influence the food people consume [17,18]. Research suggests that male and female adolescents rank differently on food literacy assessment tools. It has been suggested that female adolescents score higher on food and nutrition assessment tools because female adolescents care more about food, nutrition and health [19], have stronger concerns about their physical appearance than males [19,20], are more selective with their food choices [20,21], appear to be more interested in the general topic of food and nutrition [22], and are more confident interpreting nutrition facts tables [23]. Another factor that influences food skill acquisition in adolescents is being from a single-parent household [5], as single-parent households may experience lower incomes, higher stress, and time constraints that affect the time spent in caregiving roles [24,25].

It has been suggested that family mealtime is important as it plays a role in the development of eating behaviours for children and adolescents [26]. Fieldhouse [27] suggests that the family meal provides opportunities for parents to model healthy eating behaviours, build a balanced meal, teach about appetite and satiety, and to expose children to a variety of different food choices and tastes, all of which contribute to the development of food and nutrition knowledge. When there are healthy food options within the home environment, adolescents are more likely to have the confidence and self-efficacy to make healthier food decisions [28]. In Fitzgerald et al.’s [29] cross-sectional study examining the relationship between dietary patterns among adolescents and their level of self-efficacy, students with higher levels of self-efficacy for healthy eating consumed healthier foods, such as higher intakes of fruits and vegetables, brown bread and low-energy drinks, than those who had lower self-efficacy.

Without the knowledge needed to obtain, understand, and apply basic food and nutrition information to make healthy food choices, adolescent diets are suffering and inadequately reflecting food and nutrition recommendations for optimal health [7]. It has been suggested that developing food literacy skills, such as food and nutrition knowledge, may better equip individuals to attain healthier dietary outcomes [30]. With limited Canadian research on food and nutrition knowledge among adolescents, it is increasingly important to understand contributions to food and nutrition knowledge among this population. The objectives of this study was to determine which individual factors, sociodemographic factors, and dietary behaviours are related to food and nutrition knowledge among adolescents from the census metropolitan area (CMA) of London, ON, Canada.

## 2. Materials and Methods

### 2.1. Study Design

The data used in this cross-sectional study were baseline survey data from a larger population health intervention study conducted with high school students (i.e., 13 to 19 years of age) in the CMA of London, ON, Canada. For publicly-funded schools within the province of Ontario, the curriculum is governed by the provincial Ministry of Education. Students take a mandatory health and physical education curriculum from kindergarten to grade 8 [31]. However, after grade 8, students are only required to take one credit of health and physical education [32]. The study was approved by the University of Western Ontario’s Non-Medical Research Ethics Board and the London District Catholic School Board. Parental consent and adolescent assent were obtained prior to participation.

### 2.2. Surveys

This study used data from two baseline surveys: a parent survey and a youth survey. The parent survey consisted of 29 questions regarding food purchasing behaviours, family meal habits, and individual and family level characteristics. The parent survey was the main source for socioeconomic status (SES) data used in this study.

The youth survey included 55 items (88 questions) and consisted of 4 sections: general information; general eating habits; nutrition questions; and knowledge questions (including food knowledge and nutrition knowledge). The knowledge survey was adapted from previously validated surveys [33] and consisted of 50 questions in the format of multiple-choice questions. All guided by Canadian research conducted by the Food Literacy and Food Skills Locally Driven Collaborative Project (LDCP) working group [7,18,30], the 50 knowledge questions were categorized into two groups: food knowledge and nutrition knowledge. The Food Literacy and Food Skills LDCP working group defines food and nutrition knowledge as follows: food knowledge is understanding where one’s food comes from and what is in it, in addition to having the ability to make informed choices [30]; nutrition knowledge is understanding nutrients within foods and how they can affect one’s health [30]. Two Registered Dietitians independently assessed and categorized each knowledge question based on the above criteria to establish which type of knowledge the question was measuring. Any discrepancies between categorization were discussed until an agreement was reached.

Total knowledge score is the percentage of the 50 knowledge questions (including both the food and nutrition knowledge questions), as described in Table 1, that each student answered correctly. The food knowledge score consists of the percentage of correct answers out of the 16 items that assessed food knowledge. The nutrition knowledge score consists of the percentage of correct answers from the remaining 34 items that assessed nutrition knowledge. The knowledge scores were assessed for internal consistency using Cronbach’s Alpha [34]. The results found an alpha of 0.65 for food knowledge and an alpha of 0.75 for nutrition knowledge. These alpha values indicate that the internal consistency for food knowledge and nutrition knowledge, respectively, are weak and moderate [34].

Answering 50% or more of the survey questions (i.e., at least 25 questions) was considered a complete response. Answers were considered incorrect if they specified ‘I do not know,’ if the answer was left blank, or if they answered the question incorrectly. Students also had to have a completed parent/guardian survey. All other students were removed from analysis.

### 2.3. Covariates

The independent variables analyzed in this study are outlined in Table 2, including the level of measurement for each variable, the data source for the variables (youth or parent survey), as well as how some variables were derived using survey data.

### 2.4. Statistical Analysis

Data analysis was performed using IBM SPSS, version 26 (IBM Corp, Armonk, NY, USA). The mean and standard deviation (SD) were used to describe continuous variables and categorical variables were summarized with percentages. The median and interquartile range (IQR) was used to report the number of evenings per week that families ate dinner together.

Missing data were a result of non-response to questions either in the parent or youth survey. Results from Little’s Missing Completely at Random (MCAR) analysis revealed that missing data were MCAR. Therefore, it was appropriate to utilize multiple imputation, and more specifically fully conditional specification, to fill in the missing data for these variables [35,36]. There were 763 participants with complete data, and 205 participants had at least one independent variable that had to be imputed. A total of 1.8% of data were imputed using multiple imputation.

Three multiple linear regression models were estimated, one for each outcome variable, to test the following null hypothesis: there is no significant relationship between the knowledge scores and individual factors, sociodemographic factors, and dietary behaviours. Individual factors, sociodemographic factors, and dietary behaviours were included in each model. Collinearity diagnostics were determined through the tolerance statistic and variance inflation factor (VIF), which showed that collinearity was not a problem between covariates [37]. Only results from the imputed data models are described in this study, as the *p*-values, magnitude and direction of each predictor variable, and outcome measure do not change substantially between the two analyses. All bivariate associations between independent and dependent variables with a *p*-value < 0.10 were retained in the regression models, while key variables identified in the literature were also carried into the models. A *p*-value < 0.05 was considered statistically significant.

## 3. Results

### 3.1. Baseline Characteristics

Baseline characteristics (N = 968) are shown in Table 3. The average total score on the overall food and nutrition knowledge survey was 54.6% (SD = 14.0), while the average sub-scores were 59.8% (SD = 18.0) and 52.2% (SD = 14.0) for food knowledge and nutrition knowledge, respectively.

The average age of the participants was 15.5 years (SD = 1.2). Most were female (62.1%), Caucasian (61.9%), living in two-parent households (78.7%), and had a parent or guardian with a post-secondary education, defined as a post-secondary degree, diploma, or certificate (62.6%). The median neighbourhood family income (2016) measured at the Census Dissemination Area level was $93,677 (SD = 2755) in Canadian Dollars. Almost one-third (30.0%) of students reported using a food, nutrition, or health application, and 20.2% reported having an allergy or health condition that affected their food intake. When asked to rate their mental health, 78.4% of students reported that their mental health was good, very good, or excellent.

Students self-reported preparing or helping to prepare dinner on average 2.8 (SD = 2.0) evenings per week. Additionally, students reported that their family ate dinner together a median of 5.0 (IQR = 4.0) evenings per week. Students were asked three questions that measured attitudes towards food and nutrition. When asked to rank their agreement with the statement “I like to cook”, 64.5% of students reported that they either agreed or strongly agreed. When asked to rate the statement “Cooking or preparing meals helps me eat more healthy,” 67.0% of students reported that they either agreed or strongly agreed. Lastly, 76.2% of students either agreed or strongly agreed with the following statement “I have no problem reading and understanding food labels.”

### 3.2. Multiple Linear Regression Models

Results from the multiple linear regression models can be found in Table 4, Table 5 and Table 6. Sex was not significant in the regression models for food, nutrition, or total knowledge scores. Adolescents who identified as a visible minority scored lower on food, nutrition, and total knowledge scores than their Caucasian counterparts, 5.13%, 3.13% and 3.77% lower, respectively (*p* < 0.01). Compared to those with poor mental health, students who reported good mental health also had lower food, nutrition, and total knowledge scores, 4.35%, 3.13% and 3.55%, respectively (*p* < 0.01). It was observed that for every $10,000 increment increase in median neighbourhood family income, students scored slightly higher on food, nutrition, and total knowledge scores, 0.59%, 0.52% and 0.54%, respectively (*p* < 0.05). Adolescents who used food, nutrition, or health applications scored higher on food, nutrition, and total knowledge than students who did not use these applications, 4.32%, 2.55% and 3.11%, respectively (*p* < 0.05). Adolescents who reported confidence in reading and understanding food labels had significantly higher food, nutrition, and total knowledge scores, 7.63%, 6.36%, 6.77%, respectively. Adolescents who reported that cooking and preparing meals can help one eat healthier had significantly higher food and total knowledge scores, 3.07% and 2.13%, respectively. Lastly, adolescents who reported that they liked to cook had significantly higher nutrition and total knowledge scores, 2.07% and 2.00%.

## 4. Discussion

This study examined food and nutrition knowledge among adolescents from the CMA of London, ON, Canada, and the individual factors, sociodemographic factors, and dietary behaviours related to food and nutrition knowledge. Food literacy, including knowledge about food and nutrition, is important for adolescents to develop as higher food literacy can help them interact with our complex and evolving food system, achieve healthier dietary outcomes [11,30], and help reduce the risk of developing diet-related, non-communicable diseases over the life course [2,5,6]. This study found that knowledge scores were, on average, relatively low, with an average total knowledge score of 54.6%, and average sub-scores of 59.8% for food knowledge and 52.2% for nutrition knowledge. It is possible that lower scores were achieved for nutrition knowledge because this type of knowledge involves a deeper understanding of how the nutrients within foods can affect one’s health. These low knowledge scores are in line with previous research and suggest that adolescents may not have the necessary food and nutrition knowledge to select and consume a healthy diet [2,3,11,12,16,30].

At the cognitive level, adolescents who reported confidence in reading and understanding food labels, liking to cook, as well as having an understanding that cooking meals can lead to healthier eating had significantly higher total food and nutrition knowledge. One mechanism to explain these findings is through the capacity of dietary self-efficacy. Dietary self-efficacy focuses on building self-confidence to make healthy food choices despite possible barriers [28,40]. For example, adolescents may develop dietary self-efficacy by learning how to read and interpret food labels. Likewise, when adolescents are involved in food preparation, they develop increased dietary self-efficacy and food literacy skills [2,10,41,42,43]. It has been found that students with higher levels of dietary self-efficacy consume healthier foods, such as higher intakes of fruits and vegetables, brown bread, and low-energy drinks, than those with lower self-efficacy [3,11,29]. However, consistently throughout the literature, research suggests that a decline in preparing meals at home with family, and the absence of home economics and nutrition education in school curricula, are two of the main contributors to the observed lack of food skills and knowledge among adolescents [3,11,16,44]. Consequently, it is believed that this decline in food literacy may play a role in diet-related health outcomes such as obesity, high blood pressure, and elevated blood sugar [44]. Researchers suggest that a mandatory food and nutrition curriculum should be implemented to provide equal opportunity for adolescents to enhance their food literacy [11,44]. Taking a systems approach, implementing more food and nutrition courses into school curricula is an investment into the health and future of youth [11] and may have a large impact on the adolescent population.

The current study also found that adolescents who used food, nutrition, and health applications (i.e., smartphone ‘apps’) scored higher on food, nutrition, and total knowledge than students who did not use these applications. Given the ubiquity of smartphones and the relatively low cost of implementation compared to other approaches [45,46], nutrition interventions using a mobile application is a promising approach to teach youth about food and nutrition. Research suggests that mobile health applications can have a positive effect on the health of adolescents. For example, Dute et al. [47] examined mobile apps that promote nutrition, physical activity, and prevention of overweight in adolescents and concluded that mobile health interventions may be an effective tool for promoting healthy lifestyles. Similarly, Fedele et al. [48] conducted a meta-analysis examining if the use of mobile health interventions can improve youth health outcomes, such as a change in blood sugar or dietary intake, and found that mobile interventions resulted in a small but significant positive effect on these health outcomes. However, research is needed to better understand the effectiveness of nutrition applications as a strategy for improving food behaviour and food literacy in adolescents because many health applications focus on weight loss and food intake tracking [49,50,51], rather than food literacy. In fact, one systematic review concluded that there is a need for future research to better understand the influence that technology-driven interventions can have on changing food intake among youth [12].

Results from this study may suggest that the current food and nutrition education offered within the school setting is inadequate. Findings showed that food and nutrition knowledge only increased by 1% for every one-year increase in an adolescent’s age. Though only nutrition knowledge and total knowledge scores were statistically significant (*p* = 0.01), it can be argued that this increase is not a meaningful difference in nutritional terms. This observation may be because as a population group, Ontario high school students do not have a mandatory course dedicated to food and nutrition throughout secondary school. These findings may help bring awareness to the need for a mandatory food and nutrition course, which would empower adolescents and provide them with the opportunity to enhance their knowledge and skills related to food literacy [11,18,52]. Researchers have encouraged policy makers to implement mandatory food and nutrition curricula in the school setting as a population-level intervention to combat the decline in food literacy and improve diet-related health outcomes [8,28,37]. More research is needed to better understand food literacy as a whole construct to develop appropriate curricula for adolescents. Findings may also help guide future research towards optimal methods for delivering food literacy interventions to effectively educate teenagers and provide them with the skills to navigate the food system. From a population health perspective, future research can study how to best develop community programs that will target adolescents most at risk for low food literacy.

The call for a validated food literacy measurement tool has been consistently stated in the literature [2,5,7,12,30,53], as current studies measure different components of food literacy [2,5,8,12,14,53,54]. A validated food literacy assessment tool is critical to advance this field of study, as it would ensure accuracy in measuring the concept of food literacy across the world. Once a validated food literacy tool is published and available, it will ensure that food literacy research can be standardized, allowing for research to be comparable.

To our knowledge, this study is among the first empirical evidence for the state of food and nutrition knowledge among a Canadian adolescent population. The large sample size was also a strength as it enabled the analysis to adequately detect correlates of food and nutrition knowledge. Likewise, the youth survey was designed to measure a diverse array of content, making it possible to control for covariates in the models. However, the findings must be interpreted as correlations, as it is not possible to determine causality with cross-sectional data. It is important to acknowledge that the independent variables in all models had little explanatory power and do not explain much of the variation in the dependent variables due to the low adjusted R^2^-values. The extent to which the results are generalizable to a broader adolescent population is also unknown, as a random sample was not obtained. Nonetheless, a large sample size was achieved, and this initial sample of youth does provide important insight into the current state of food and nutrition knowledge among a diverse group of adolescents.

This study fills a gap in the literature as it provides a better understanding of, and correlates of, food and nutrition knowledge among adolescents. Findings from this study may help guide policy makers, researchers, and public health professionals in developing appropriate food and nutrition programs and curriculums to enhance food literacy among adolescents.

## Figures and Tables

**Table 1:** Knowledge Survey Questions Coded as Food or Nutrition Knowledge.

Food Knowledge Questions
Do you think these foods and drinks are typically high or low in added sugar?
Do you think these foods are typically high or low in salt?
Which of these foods has the most trans-fat?
Compared to minimally processed foods, [what nutrient is higher/lower in] processed foods?
If a person wanted to buy a yogurt at the supermarket, which would have the least sugar/sweetener?
Looking at product 1, what are the sources of sugar in the ingredient list?
[Agree or Disagree]. If the % Daily Value for sodium was greater than 15%, it is considered “high in” sodium.
[Agree or Disagree]. “Light” foods (or diet foods) are always good options because they are low in calories.
**Nutrition Knowledge Questions**
How many servings of fruit and vegetables per day do experts advise teens to eat as a minimum?
Do health experts recommend that people should be eating more, the same amount, or less of the following items?
How many times per week do experts recommend that people eat fish (e.g., salmon, tuna, tilapia)?
How many times per week do experts recommend that people eat breakfast?
Do you think these foods are typically high or low in fibre?
Do you think these foods are a good source of protein?
[How does the] amount of calcium in a glass of whole milk compare to a glass of skimmed milk?
Which would be the healthiest and most balanced sandwich lunch?
Which would be the healthiest burger choice when eating at a restaurant?
Looking [at the labels] for products 1 and 2, which one has the most calories (kcal) per biscuit?
Which of these diseases is related to a low intake of fibre?
Which of these diseases is related to how much sugar people eat?
Which of these diseases is related to how much salt (or sodium) people eat?
Which one of these foods is classified as having a high Glycemic Index?
[Agree or Disagree]. To maintain a healthy weight people should cut fat out completely.

Note: The questions were asked in a multiple-choice format with 2 to 4 options to choose from for each question.

**Table 2:** Definition of Independent Variables.

Variable Name	Data Type	Data Source	Measurement
Visible Minority	Binary	Youth survey and parent survey	Visible Minority (1): South Asian, East Asian, Middle Eastern, Latin American, Indigenous, Black, and Other. Caucasian (0): White/Caucasian
Male	Binary	Youth survey	Male (1); Female (0). Students self-reported their gender. Four students identified as non-binary were considered missing.
Age	Continuous	Youth survey	Age in years
Allergy or Health Condition	Binary	Youth survey	Yes (1); No (0). Derived from 2 binary questions: (1) Do you have health conditions that affect your eating patterns? (2) Do you have food allergies and/or intolerances that affect your eating patterns?
Use Food, Nutrition, Health Apps	Binary	Youth survey	Yes (1); No (0). Measured with the question: Do you use any food, nutrition, or health apps on your smartphone or tablet?
Eating with Family	Continuous	Youth survey	Number of nights per week. Measured with the question: During a typical week, how many evenings do you eat dinner with your family?
Prepare Dinner	Continuous	Youth survey	Number of nights per week. Measured with the question: During a typical week, how many evenings do you prepare or help prepare dinner?
Median Neighbourhood Family Income	Continuous	Parent survey	$10,000 Canadian dollars. Median family income for the dissemination area (DA) of the primary home postal code. DAs are frequently used as proxies for neighbourhoods. [38]
Lone Parent Household	Binary	Parent survey	Yes (1); No (0)
Maximum Education	Categorical	Parent survey	Post-Secondary Education (reference): Trade or other non-university certificate or diploma, University certificate or diploma below bachelor’s level, and bachelor’s degree. High School Diploma or Less: Less than high school diploma or equivalent and high school diploma or equivalency. Post-Graduate Education: University certificate, diploma, or degree above the bachelor’s level. Derived from the maximum education level of all parents/guardians who live in the primary household.
Good Self-Reported Mental Health	Binary	Youth survey	Good Mental Health (1): Good, Very Good, and Excellent Poor Mental Health (0): Poor and Fair Measured with a 5-pt Likert scale question: In general, how do you rate your own mental health?
Food Label Confidence	Binary	Youth survey	Agree (1): Agree and Strongly Agree Disagree (0): Neutral, Disagree, and Strongly Disagree Measured with a 5-pt Likert scale question: I have no problem reading and understanding food labels
Prepare Meals	Binary	Youth survey	Agree (1): Agree and Strongly Agree Disagree (0): Neutral, Disagree and Strongly Disagree Measured with a 5-pt Likert scale question: Cooking or preparing meals helps me eat more healthy
Enjoy Cooking	Binary	Youth survey	Agree (1): Agree and Strongly Agree Disagree (0): Neutral, Disagree and Strongly Disagree Measured with a 5-pt Likert scale question: I like to cook

**Table 3:** Characteristics of the Sample (N = 968).

Characteristics	Total Sample (Complete Case)
Knowledge Scores, mean (SD) ^†^	
Food knowledge score	59.8 (18.0)
Nutrition knowledge score	52.2 (14.0)
Total knowledge score	54.6 (14.0)
Individual Characteristics	
Visible minority, n ^‡^ (%)	369 (38.1)
Male, n ^‡^ (%)	362 (37.9)
Missing cases	12 (1.2)
Age in years, mean (SD) ^†^	15.5 (1.2)
13 years, n ^‡^ (%)	21 (2.2)
14 years, n ^‡^ (%)	231 (23.9)
15 years, n ^‡^ (%)	225 (23.2)
16 years, n ^‡^ (%)	268 (27.7)
17 years, n ^‡^ (%)	186 (19.2)
18 years, n ^‡^ (%)	33 (3.4)
19 years, n ^‡^ (%)	4 (0.4)
Good self-reported mental health, n ^‡^ (%)	758 (78.4)
Sociodemographic Characteristics	
Lone parent household, n ^‡^ (%)	206 (21.3)
Missing cases	19 (2.0)
Maximum Education, n ^‡^ (%)	
High school diploma or less	117 (12.1)
Post-secondary degree, diploma, certificate	606 (62.6)
Post-graduate degree education	177 (18.3)
Missing cases	68 (7.0)
Median neighbourhood family income, mean of median (SD) ^†^	^§^ 9.37 (0.28)
Food Behaviours and Attitudes	
Number of nights/week student helps prepare dinner, mean (SD) ^†^	2.8 (2.0)
Missing cases, n ^‡^ (%)	54 (6.6)
Number of nights/week family eats dinner together, median (IQR)	5.0 (4.0)
Missing cases, n ^‡^ (%)	34 (3.5)
Use food, nutrition, or health apps, n ^‡^ (%)	290 (30.0)
Allergy or health condition affecting food intake, n ^‡^ (%)	196 (20.2)
Missing Cases	54 (6.6)
Confidence in reading and understanding food labels, n (%)	738 (76.2)
Cooking helps me to be healthy, n ^‡^ (%)	649 (67.0)
I like to cook, n ^‡^ (%)	625 (64.5)

N, total sample. ^†^ SD, standard deviation. ^‡^ n, number of cases. ^§^ Median neighbourhood family income is presented in Canadian dollars and by tens of thousands of dollars ($10,000 CAD is approximately $6929 USD [39]).

**Table 4:** Multiple Linear Regression for Food Knowledge Score (N = 968).

	Model 1: Imputed Data				
	β *	SE ^†^	*p* ^‡^	CIs ^§^ (0.95)	
**Individual Characteristics**					
Visible minority	−5.53	1.29	<0.01	−8.05	−3.00
Male	0.56	1.26	0.66	−1.92	3.04
Age (in years)	0.94	0.51	0.06	−0.06	1.93
Good self-reported mental health	−4.38	1.53	<0.01	−7.39	−1.37
**Sociodemographic Characteristics**					
Lone parent household	−0.41	1.50	0.78	−3.35	2.53
Median neighbourhood family income ($10,000 CAD)	0.3	0.24	0.34	−0.24	0.69
**Parent education Level (ref: post-secondary education)**					
High school or less	−2.03	1.89	0.28	−5.75	1.68
Post-graduate education	3.00	1.51	0.05	0.04	5.96
**Food Behaviours and Attitudes**					
Number of nights/week student helps prepare dinner	−0.85	0.33	0.01	−1.49	−0.21
Number of nights/week family eats dinner together	0.17	0.31	0.58	−0.43	0.77
Use food, nutrition, health apps	3.34	1.31	0.01	0.76	5.92
Allergy/health condition affecting food intake	2.02	1.47	0.17	−0.87	4.92
Confidence in reading and understanding food labels	5.75	1.44	<0.01	2.94	8.57
Cooking and preparing meals helps me eat healthier	2.95	1.34	0.03	0.31	5.59
I like to cook	2.24	1.32	0.09	−0.35	4.84
(Constant)	34.65	9.58	<0.01	15.84	53.46
Adjusted *R*^2^-value	0.089				

* β, coefficient. ^†^ SE, standard error. ^‡^ *p*, *p*-value. ^§^ CIs, confidence intervals.

**Table 5:** Multiple Linear Regression for Nutrition Knowledge Score (N = 968).

	Model 2: Imputed Data				
	β *	SE ^†^	*p* ^‡^	CIs ^§^ (0.95)	
**Individual Characteristics**					
Visible minority	−3.86	0.99	<0.01	−5.80	−1.91
Male	−1.66	0.97	0.09	−3.56	0.25
Age (in years)	1.06	0.39	0.01	0.30	1.82
Good self-reported mental health	−3.48	1.18	<0.01	−5.79	−1.17
**Sociodemographic Characteristics**					
Lone parent household	−1.94	1.15	0.09	−4.20	0.32
Median neighbourhood family income ($10,000 CAD)	0.38	0.18	0.04	0.03	0.73
Parent education level (ref: post-secondary education)					
High school or less	−3.59	1.46	0.01	−6.44	−0.73
Post-graduate education	3.21	1.16	0.01	0.93	5.48
**Food Behaviours and Attitudes**					
Number of nights/week student helps prepare dinner	−0.65	0.25	0.01	−1.1	−0.16
Number of nights/week family eats dinner together	0.12	0.24	0.62	−0.35	0.58
Use food, nutrition, health apps	1.62	1.01	0.11	−0.37	3.60
Allergy/health condition affecting food intake	1.18	1.13	0.30	−1.04	3.40
Confidence in reading and understanding food labels	5.14	1.10	<0.01	2.97	7.30
Cooking and preparing meals helps me eat healthier	1.11	1.03	0.29	−0.92	3.13
I like to cook	1.96	1.02	0.05	−0.04	3.95
(Constant)	27.71	7.37	<0.01	13.25	42.18
Adjusted *R*^2^-value	0.120				

* β, coefficient. ^†^ SE, standard error. ^‡^ *p*, *p*-value. ^§^ CIs, confidence intervals.

**Table 6:** Multiple Linear Regression for Total Knowledge Score (N = 968).

	Model 3: Imputed Data				
	β *	SE ^†^	*p* ^‡^	CIs ^§^ (0.95)	
**Individual Characteristics**					
Visible minority	−4.39	0.98	<0.01	−6.32	−2.46
Male	−0.95	0.96	0.33	−2.84	0.95
Age (in years)	1.02	0.39	0.01	0.26	1.77
Good self-reported mental health	−3.77	1.16	<0.01	−6.06	−1.48
**Sociodemographic Characteristics**					
Lone parent household	−1.45	1.14	0.21	−3.69	0.79
Median neighbourhood family income ($10,000 CAD)	0.33	0.18	0.07	−0.02	0.68
Parent education level (ref: post-secondary education)					
High school or less	−3.09	1.44	0.03	−5.92	−0.26
Post-graduate education	3.14	1.15	<0.01	0.88	5.40
**Food Behaviours and Attitudes**					
Number of nights/week student helps prepare dinner	−0.72	0.25	<0.01	−1.20	−0.23
Number of nights/week family eats dinner together	0.13	0.23	0.57	−0.33	0.59
Use food, nutrition, health apps	2.17	1.00	0.03	0.20	4.14
Allergy/health condition affecting food intake	1.45	1.12	0.120	−0.76	3.66
Confidence in reading and understanding food labels	5.33	1.10	<0.01	3.18	7.48
Cooking and preparing meals helps me eat healthier	1.70	1.03	0.10	−0.32	3.71
I like to cook	2.05	1.01	0.04	0.07	4.03
(Constant)	29.93	7.31	<0.01	15.59	44.28
Adjusted *R*^2^-value	0.128				

* β, coefficient. ^†^ SE, standard error. ^‡^ *p*, *p*-value. ^§^ CIs, confidence intervals.

## Data Availability

Ethics agreements prohibit the research team from making the data publicly available.
